# Posttraumatic Growth and Resilience: Their Distinctive Relationships with Optimism and Pessimism

**DOI:** 10.3390/bs15111519

**Published:** 2025-11-08

**Authors:** Kanako Taku, Amber Efthemiou

**Affiliations:** 1 Department of Psychology, Oakland University, Rochester, MI 48309, USA; aefthemi@emich.edu; 2 Department of Psychology, Eastern Michigan University, Ypsilanti, MI 48197, USA

**Keywords:** posttraumatic growth, resilience, optimism, pessimism, cognitive expectancy, belief update

## Abstract

Posttraumatic growth (PTG) and resilience should have distinct features due to their theoretical background, and yet their respective relationships with optimism have been consistently positive. Their relationships with pessimism have been understudied, which obscures how PTG and resilience may conceptually differ. We hypothesize that the differences may emerge whether optimism and pessimism are evaluated as cognitive expectancies or dispositional personality traits. The current study examined how optimism and pessimism would be distinctly associated with PTG and resilience, depending on whether optimism and pessimism reflect dispositional personality traits or cognitive expectancies. Midwestern United States university students (*N* = 347) completed an in-person survey that included measures examining optimism and pessimism as personality traits and a cognitive task estimating the likelihood of positive and negative future events happening to them and happening to others and re-estimating after obtaining novel information (i.e., belief update), in addition to PTG and resilience. Results indicated that dispositional optimism was positively associated with both PTG and resilience, whereas dispositional pessimism was negatively associated with only resilience. Furthermore, higher expectancy of positive events to be happening in the future was mostly associated with PTG whereas lower expectancy of negative events to be happening in the future was mostly associated with resilience. In addition, the perception that positive events would be more likely to happen to them than to others was only associated with resilience. Findings regarding the relationships with adjusted cognitive expectancies (i.e., belief update) were mixed. The current findings reveal potential distinctions between PTG and resilience by highlighting that they may have asymmetrical relationships with optimism and pessimism, depending on whether optimistic/pessimistic characteristics are considered as personality traits or cognitive expectations of positive and negative future events.

## 1. Introduction

Ever since the concept of posttraumatic growth (PTG: Tedeschi & Calhoun, 1996) appeared in the field, debates have existed as to how it may differ from resilience, because both include some positive outcomes after experiencing some negative events. Specifically, PTG refers to “positive psychological change experienced as a result of the struggle with highly challenging life circumstances” (Tedeschi & Calhoun, 2004, p. 1). Throughout the process initiated by highly challenging life circumstances, an individual may experience psychological struggle, which includes reexamining one’s core values, ruminating on the event, and coping with the challenges and distress, according to the PTG theoretical model (Tedeschi et al., 2018). Following this process, individuals may then gain a greater appreciation for life, an increased sense of personal strength, realize new possibilities, experience a spiritual and existential change, or relate to others more, which can be conceptualized as the five domains of PTG (Tedeschi & Calhoun, 1996). On the other hand, resilience, although it has various definitions, typically refers to the emergence of favorable adjustment in the face of chronically aversive circumstances and also shows a healthy trajectory even after experiencing acute stressful life events (Bonanno & Diminich, 2013), demonstrating the “ability to bounce back or recover from stress” (Smith et al., 2008, p. 195). With these definitions, the two constructs are different, as “resilience refers to maintaining or returning to normal functioning without exhibiting elevated pathological outcomes, while growth entails a change for the better following adversity” (Chen & Wu, 2017, p. 23). Conceptually, it is more likely for people to have more resilient tendencies when they experience PTG than for people to experience PTG because they had more resilient tendencies, as “resilient outcomes may provide little need or opportunity for posttraumatic growth” (Westphal & Bonanno, 2007, p. 425).

With these conceptual and empirical differences, one would expect PTG and resilience to have distinct features and thus should show different patterns of associations with other psychological constructs, such as optimism. And yet, their respective relationships with optimism have been consistently positive (Schaefer et al., 2018; Zlotnick & Manor-Lavon, 2023). Moreover, because pessimism is conceptually the opposite of optimism, one would expect the similar patterns of associations that PTG and resilience may have with pessimism, namely, consistently negative. However, that is not the case. For example, a study focusing on the psychological changes in outlook after the COVID-19 pandemic (Jurišová et al., 2023) revealed that, even though perceived positive changes were positively associated with optimism, and optimism was negatively associated with pessimism, positive changes showed no association with pessimism. Similar findings have been obtained in meta-analytical results (Turner et al., 2017) which strengthen the argument that optimism and pessimism are not always opposites. And yet, resilience was consistently negatively associated with pessimism (Oh et al., 2024), except in one study that identified pessimism as a significant “positive” predictor of resilience (Shaheen et al., 2023), which overall obscures how PTG and resilience may conceptually differ in relation to optimism and pessimism.

One possibility is that all studies that were reviewed above conceptualized optimism and pessimism as personality or dispositional traits rather than cognitive expectancies of positive or negative life events to be happening in the future. This is important because, even though optimism and pessimism are conceptually the opposite, if one would feel that bad things could happen any time, it may suppress resilience but may not be relevant to PTG as realization of personal growth includes acceptance of the new reality that is not always positive (Tedeschi et al., 2018). Furthermore, the fact that the number of articles that discuss the relationships between optimism and PTG or resilience is higher than those discussing the relationships between pessimism and PTG or resilience may demonstrate that more researchers consider experiencing personal growth or becoming more resilient and becoming more optimistic to be synonymous or at least optimism to be more relevant than pessimism. Even clinical suggestions are made such that “interventions aimed at increasing optimism, social support, and specific coping strategies may promote positive changes in the aftermath of trauma” (Prati & Pietrantoni, 2009, p. 379), without mentioning if intervention to be less pessimistic might be necessary, being argued decades ago (Robinson-Whelen et al., 1997). In sum, the current literature does not provide comprehensive understanding of the role of optimism/pessimism in PTG and resilience in not only theoretical perspectives but applied practice.

To address this issue, we designed the current study to understand the conceptual differences between PTG and resilience by investigating their distinctive associations with optimism and pessimism as cognitive patterns, in addition to personality dispositional traits. Examining optimism and pessimism as cognitive patterns conceptualizes them on a continuum in relation to certain life events rather than fixed, dispositional traits (Hecht, 2013). Traditionally, “optimists are people who tend to hold positive expectancies for their future; pessimists are people who tend to hold more negative expectations for the future” (Scheier et al., 1994, p. 1063). In other words, people with optimistic tendencies should anticipate positive events are likely to happen to them and negative events are less likely to happen to them in the future. Similarly, people with pessimistic tendencies should anticipate positive events are less likely to happen to them and more negative events are likely to happen to them in the future.

The aforementioned conceptualization of optimism and pessimism as cognitive patterns should shed light onto their respective roles as a factor in explaining distinctions between PTG and resilience, because it measures cognitive aspect that is different from self-image, that is, dispositional personality traits (i.e., the degree to which they believe themselves to be optimistic or pessimistic). We, thus, hypothesize that people who anticipate that positive events are more likely to happen to them would report higher PTG and resilience, because if one can hold such positive cognitive expectancies, they are also more likely to show resilient tendencies and recognize how much they changed in a positive way after experiencing a highly stressful life event, known as PTG. On the other hand, people who believe negative events are less likely to happen to them would show higher resilient tendencies. However, we are unable to formulate specific hypotheses regarding the role of cognitive expectancies of future negative life events in PTG, because such expectancies may indicate acceptance of reality (i.e., coexistence of positive and negative) but may also indicate a pessimistic worldview; thus, possibly canceling each other out.

In addition, just because one assumes positive events are likely to happen to them, it does not mean if they also believe the positive events are likely to happen to other people as well. This kind of cognitive contrast or cognitive bias is called comparative optimism, which refers to the tendency to believe that positive events are more likely to happen to them compared to others, whereas negative events are more likely to happen to others compared to themselves (Otten & van Der Pligt, 1996). Conversely, comparative pessimisms refers to the opposite (i.e., negative events are more likely to happen to oneself compared to others, and positive events are more likely to happen to others compared to oneself). We hypothesize that people who show comparative optimism are more likely to show resilient tendencies, because resilience is closely related to social competence (Garcia & Berzenski, 2023) and is positively associated with sense of confidence (Howell et al., 2021). However, comparative optimism may or may not be associated with PTG because comparative optimism requires social comparisons between oneself and others, but such mental comparisons may be irrelevant to PTG. This hypothesis is exploratory, as there are no previous studies.

Finally, if one is able to update and adjust their beliefs (e.g., anticipated likelihood of positive events or negative events happening to them in the future) when they obtain novel information about the average likelihood, especially if their original estimations were different from the average, they may show more resilient tendencies, because individuals with highly resilient tendencies are expected to be flexible (Koole et al., 2015). A question then arises, if people choose not to update their beliefs even when they realize that their original estimation was “incorrect”, do they show PTG? Previous studies indicate that there are always some people who show no sign of updating (Karnick et al., 2024; Sauvayre, 2017), but because the purpose of most studies using belief updates is to identify people who “updated”, less attention has been given to those who show no change. We, therefore, hypothesize that individuals who show changes in their cognitive expectancies are likely to report resilient tendencies due to their flexible mindset. However, it is possible that, depending on the event they expect (e.g., having a successful career), people who show no change may report a high level of resilience due to their confidence (He et al., 2013) rather than less flexible or biased attitudes. On the other hand, whether people update their beliefs or not may have no impact on PTG; however, due to the lack of past studies examining this phenomenon, it is difficult to propose a set of concrete hypotheses.

In sum, the current study investigates how optimism and pessimism are associated with PTG and resilience, depending on whether optimism and pessimism reflect personality dispositional traits or cognitive expectancies (i.e., events that are likely to happen to themselves, themselves compared to others, and belief updates when they obtain novel information regarding potential future life event occurrences among average people). We present the following hypotheses, which are exploratory in nature, due to the lack of enough evidence in the literature, which in turn may show the originality of the current study. As such, demonstrating how optimism and pessimism may be associated with PTG and resilience, depending on how they are conceptualized as personality trait or cognitive expectancies, may contribute to our knowledge of the distinction between PTG and resilience:

**Hypothesis 1.**  *Dispositional optimism would be positively correlated with PTG and resilience, whereas dispositional pessimism would be negatively correlated with resilience but may or may not be correlated with PTG. Cognitively, on the other hand, people who believe positive events are likely to happen to them would show higher PTG and resilience, whereas people who believe negative events are less likely to happen to them would show higher resilience but may or may not show a higher tendency in reporting PTG.*

**Hypothesis 2.**  *People who show comparative optimism would show resilient tendencies but may or may not be associated with PTG.*

**Hypothesis 3.**  *People who update their beliefs when they obtain novel information may show resilient tendencies due to their flexibility; however, depending on the event, people who “stick to” their beliefs may also show resilient tendencies due to their competence as well as sense of confidence. Further, updating of beliefs may or may not be associated with PTG.*

## 2. Method

### 2.1. Participants

A total of 347 university students, 278 females, 68 males, and 1 preferring not to answer, were recruited from the Midwestern region of the US to participate in the pencil-and-paper study, conducted in 2019. Their mean age was 20.08 (*SD* = 4.54). Table 1 presents complete demographics.

### 2.2. Measures

Posttraumatic Growth. A short form of the PTG Inventory (PTGI-SF: Cann et al., 2010) was used to measure the degree to which people perceive positive changes that they experienced after a highly stressful/challenging and potentially traumatic life event that they had experienced and found most impactful in the past five years. If participants did not report a highly stressful or potentially traumatic in the past five years, they were asked to compare themselves to who they were five years ago as to still account for personal growth; however, these participants were not included in the current analyses. The PTGI-SF consists of 10 items (e.g., “I have a greater appreciation for the value of my own life”). Participants rated each of the items using a six-point Likert scale ranging from 0, “I did not experience this change as a result of my crisis” to 5, “I experienced this change to a very great degree as a result of my crisis”. Cronbach’s alpha coefficient was reported as 0.86 (Cann et al., 2010). For the current sample, it was 0.85.

Resilience. The Brief Resilience Scale (Smith et al., 2008) was used to measure the level of resilience, assessing the individual’s perception of their own ability to bounce back and recover following adversity. This scale includes 6 items (e.g., “I tend to bounce back quickly after hard times”). Of them, 3 items were reverse coded (e.g., “I have a hard time making it through stressful events”). The participants were asked to indicate the extent of their agreement with statements on a five-point Likert scale ranging from 1, “strongly disagree” to 5, “strongly agree”. Cronbach’s alpha was reported, ranging from 0.80 to 0.91, depending on samples (Smith et al., 2008). For the current sample, it was 0.86.

Optimism and Pessimism as Dispositional Traits. The Revised Life Orientation Test (Scheier et al., 1994) was used to measure the levels of dispositional optimism and pessimism. The scale includes 10 items. Of them, 3 items assess optimism (e.g., “In uncertain times, I usually expect the best”), 3 items assess pessimism (“If something can go wrong for me, it will”), and 4 are filler items. The participants were asked to indicate the extent of their agreement with statements on a five-point Likert scale ranging from 0, “strongly disagree” to 4, “strongly agree”. Although some studies reverse code the pessimism subscale and report one overall score (e.g., Scheier et al., 1994), the current study looked at each subscale (i.e., optimism and pessimism) separately. Psychometric evaluations of this scale have reported Cronbach’s alphas ranging from 0.59 to 0.72 and 0.51 to 0.74 for the optimism and pessimism subscales, respectively (Hinz et al., 2022; Pan et al., 2017). Cronbach’s alpha for the current sample was 0.71 for the optimism subscale and 0.81 for the pessimism subscale.

Optimism and Pessimism as Cognitive Patterns. The Judgment of Life Events Scale (Zelenski & Larsen, 2002) was used to assess optimistic and pessimistic cognitive judgment by asking the participants to estimate the likelihood of positive and negative future events to happen to them (i.e., optimism and pessimism as a cognitive pattern or expectation). Although the original scale includes 12 positive events (e.g., “what are the chances that you will get a 4.0 for a semester?”) and 12 negative events (e.g., “what are the chances that you will get HIV or AIDS?”), we used a modified version with 10 events each (e.g., “what are the chances that you will receive an A in a class?”; “what are the chances that you will develop a brain tumor?”) to make the events more relevant to our current sample. Research assistants who identified as members of our target sample (i.e., undergraduate students) piloted this measure to address content validity and clarity where they provided the likelihood of events for each of the 20 events included. We then asked the participants to indicate the likelihood that each of the events happens in the future to someone of a similar age and gender to assess comparative optimism. Reliability analyses of this scale have reported a Cronbach’s alpha of 0.74 for the positive life events and 0.82 for the negative life events (Gherasim et al., 2016). For the current sample, Cronbach’s alpha of the positive events was 0.65 for the self (first estimate) and 0.70 comparatively (second estimate), and Cronbach’s alpha of the negative events was 0.71 for the self (first estimate) and 0.82 comparatively (second estimate).

Belief Update. The paradigm of the Belief Update Task (Sharot et al., 2011) was used to assess if and how participants would be willing to change their estimated likelihood of each event that they provided when they receive novel information. We presented the participants a list of “actual likelihood” estimates that we prepared based on a pilot study right after they completed the Judgments of Life Events Scale that is mentioned above. The pilot data was calculated by the mean score of research assistants’ event likelihood estimations and we decreased this likelihood by 10 percent for odd numbered events and increased this likelihood by 10 percent for even numbered events as to provide a randomized balance of modified estimations for use in the task. As the participants reviewed the “actual likelihood” estimates, which were contextually described as the “likelihood” of happening to the average person, they were asked to reevaluate the likelihood of the same positive and negative events happening to them in the future (third estimate). In the current study, we used a total of 20 events to reduce the participants’ burden, unlike the original study that recommends including at least 40 trials or preferably up to 80 trials (Sharot & Garrett, 2022). Table 2 shows the list of events and the “actual likelihoods” we presented to the participants. For example, the participants overall estimated they had an 83.73% chance to receive an A in a class; thus, when they saw the “actual likelihood” presented after their initial estimation, many participants might have felt that they were too optimistic, because it was 66%; thus, many of them updated their estimate and, in general, provided a lower estimate (e.g., 77.45%) after seeing the “actual likelihood”.

### 2.3. Procedure

Participants were recruited from a university’s subject pool system. Upon completion of the study, participants received course credit. After reading and signing the consent form, participants provided demographic information and then completed the measures described above. When responding to PTG Inventory, they reported highly stressful or potentially traumatic life events that happened during the past five years and then selected the most impactful event if they experienced multiple events. Finally, they completed a cognitive task that includes belief updates.

### 2.4. Data Analysis

Hypothesis 1 regarding the respective relationships that optimism and pessimism have with PTG and resilience depending on whether optimism/pessimism are measured as dispositional personality traits or cognitive patterns was tested using correlations. Due to the inter-correlations between dispositional optimism and pessimism, multiple regression analyses were also conducted. Due to the variabilities among the events, correlations were obtained with cognitive expectancy for each event individually.

Hypothesis 2 regarding the roles of comparative optimism in PTG and resilience was tested using one-way ANOVAs with Tukey post hoc tests. Specifically, participants were categorized into three groups for each event by comparing the initial estimate (i.e., the likelihood of the event happening to themselves) and the second estimate (i.e., the likelihood of the event happening to others; comparative optimism and pessimism). The first group refers to those who showed comparative optimism (i.e., provided a second estimate that was lower than the first for positive events; provided a second estimate that was higher than the first for negative events). The second group refers to those who provided the same estimates for them and for others. Finally, the third group refers to those who showed comparative pessimism (i.e., provided a second estimate that was higher than the first for positive events; provided a second estimate that was lower than the first for negative events).

Hypothesis 3 regarding the role of the willingness to update the beliefs (i.e., change the estimated likelihood) in PTG and resilience was tested using one-way ANOVAs with Tukey post hoc tests. Specifically, participants were categorized into four groups for each event by comparing the first estimate (for themselves) and the third estimate (for themselves again but while reviewing the “actual likelihood”). Individuals labeled (1) “Optimistic/No Change” or (2) “Pessimistic/No Change” refer to those who indicated themselves more or less likely to experience a certain event than the given likelihood, then made no adjustment after reviewing the actual likelihood. Individuals labeled (3) “Optimistic/Changed” or (4) “Pessimistic/Changed” refer to those who first indicated themselves more or less likely to experience a certain event than the given likelihood, then made an adjustment when they reviewed the actual likelihood. Participants who happened to give the same ratings as the “actual likelihood” were excluded from these analyses.

The data were analyzed using SPSS 27. To avoid inflating Type I errors, a significance level was set at 1% for the main results and 5% for the follow-up post hoc comparisons in case the main results were significant.

## 3. Results

### 3.1. Descriptive Statistics

The mean amount of highly stressful or potentially traumatic events participants experienced in the past five years was 4.24 (*SD* = 2.21) with five participants of the overall sample reporting no highly stressful or potentially traumatic events who were excluded from the analyses. The overall mean of the PTG was 3.20 (*SD* = 1.04) and the resilience was 3.05 (*SD* = 0.83). PTG and resilience were not correlated, *r* = −0.02. The mean of dispositional optimism was significantly higher (*M* = 3.31, *SD* = 0.91) than the mean of dispositional pessimism (*M* = 2.75, *SD* = 0.98), *t*(345) = 6.19, *p* < 0.001, Cohen’s *d* = 1.69. Dispositional optimism and pessimism were negatively associated with each other, *r* = −0.58, CI_95%_ [−0.65, −0.51], *p* < 0.001. As shown in Table 2, the initial estimate of the likelihood of each positive/negative event happening to them varied considerably.

### 3.2. Descriptive Statistics and Relationships Between Variables: Hypothesis 1

Dispositional optimism and PTG as well as resilience were both positively correlated, *r* = 0.32, CI_95%_ [0.23, 0.41], *p* < 0.001, and *r* = 0.33, CI_95%_ [0.23, 0.42], *p* < 0.001, respectively. Dispositional pessimism and resilience were negatively correlated, *r* = −0.36, CI_95%_ [−0.45, −0.26], *p* < 0.001, however, dispositional pessimism and PTG were not, *r* = −0.10 (Table 3). Multiple regression analyses (Table 4) revealed that dispositional optimism and pessimism contributed to an overall model explaining PTG, *F*(2, 344) = 22.16, *p* < 0.001, adjusted R^2^ = 0.11, with dispositional optimism, *β* = 0.40, *p* < 0.001, as the only significant and positive predictor. For resilience, dispositional optimism and pessimism contributed to an overall model, *F*(2, 340) = 29.89, *p* < 0.001, adjusted R^2^ = 0.15, with dispositional optimism, *β* = 0.18, *p* = 0.004 as a positive, significant predictor, and dispositional pessimism, *β* = −0.26, *p* < 0.001, as a negative, significant predictor. In addition, the correlations between the initial estimates of each of the 20 events and PTG as well as resilience were obtained, revealing primarily positive correlations between positive events and PTG, whereas there were negative correlations between negative events and resilience (Table 5). These patterns partially support Hypothesis 1.

### 3.3. Comparative Optimism/Pessimism and PTG and Resilience: Hypothesis 2

Three groups (i.e., those who showed comparative optimism, those who provided the same estimates, and those who showed comparative pessimism) were compared to test Hypothesis 2. A series of one-way ANOVAs indicated that, of twenty events, one event (i.e., “Control my weight for at least 10 years”) yielded significant differences in the level of resilience, *F*(2, 336) = 5.52, *p* = 0.004, but none for PTG. Specifically, people (*n* = 173) who showed comparative optimism (i.e., the mean for their initial estimate was 84.71 with *SD* = 14.87 and the mean for their second estimate was 55.25 with *SD* = 19.73) held significantly more resilient tendencies (*M* = 3.18, *SD* = 0.84) than those (*n* = 86) who showed comparative pessimism (i.e., the mean for their initial estimate was 25.17 with *SD* = 22.74 and the mean for their second estimate was 51.87 with *SD* = 24.42; their resilience score was *M* = 2.83, *SD* = 0.76), at *p* = 0.004. There were no significant differences in the levels of resilience between these two groups and those who gave the same estimates for themselves and others (*n* = 82, their initial and second estimates were *M* = 57.20, *SD* = 25.68).

### 3.4. Roles of Belief Update Group Differences in PTG and Resilience: Hypothesis 3

A series of one-way ANOVAs were conducted with four groups on the levels of PTG and resilience. Of twenty events, three yielded significant results.

First, one of the ten positive events (i.e., “Never have any financial problems for the rest of life”) yielded significant differences in PTG, *F*(3, 322) = 5.06, *p* = 0.002, η^2^ = 0.05, but none for resilience. Specifically, people (*n* = 95) who were initially optimistic but then adjusted their estimates after seeing the actual likelihood was “30%” (i.e., the mean for their initial estimate was 60.35 with *SD* = 18.52 and the mean for their third estimate was 30.26 with *SD* = 19.28) reported significantly higher PTG (*M* = 3.51, *SD* = 0.90) than those (*n* = 170) who were initially pessimistic but then adjusted after seeing the actual likelihood was “30%”, at *p* = 0.005, as well as those (*n* = 49) who were initially pessimistic and did not adjust, at *p* = 0.012, by using Tukey HSD post hoc comparisons (Table 6).

Second, another positive event (i.e., “Live with partner happily ever after”) also yielded significant differences in PTG, *F*(3, 339) = 4.06, *p* = 0.007, η^2^ = 0.04, but none for resilience. Specifically, people (*n* = 126) who were initially optimistic but then adjusted after seeing the actual likelihood was “63%” reported significantly higher PTG (*M* = 3.39, *SD* = 0.97) than those (*n* = 100) who were initially pessimistic and then adjusted at *p* = 0.026, by using Tukey HSD post hoc comparisons (Table 7). In addition, those who were initially optimistic and then adjusted (*n* = 126) reported higher PTG than those who were initially pessimistic and did not make changes when they saw the actual likelihood was “63%” (*n* = 33, their initial and third estimates were *M* = 42.73, *SD* = 15.67) at *p* = 0.037.

Third, another positive event (i.e., “Control my weight for at least 10 years”) yielded significant differences in resilience, *F*(3, 335) = 4.48, *p* = 0.004, η^2^ = 0.04, but none for PTG. Specifically, people (*n* = 70) who were initially optimistic and did not update their beliefs after seeing the actual likelihood as “81%” (i.e., the means for their initial and the third estimates were 94.41 with *SD* = 5.52) held significantly more resilient tendencies (*M* = 3.30, *SD* = 0.81) than those (*n* = 169) who were initially pessimistic but then changed their estimates (i.e., the mean for their initial estimate was 42.44 with *SD* = 26.46 and the mean for their third estimate was 63.59 with *SD* = 23.94; their resilience score was *M* = 2.95, *SD* = 0.79) at *p* = 0.014, by using Tukey HSD post hoc comparisons (Table 8). Also, those who were initially optimistic and did not change (*n* = 70) held significantly more resilient tendencies than those who were initially pessimistic and did not adjust (*n* = 52) (i.e., the means for their initial and third estimates were *M* = 55.88, *SD* = 24.06; their resilience score was *M* = 2.87, *SD* = 0.85), at *p* = 0.021.

## 4. Discussion

The purpose of the current study was to investigate the distinctions between PTG and resilience by exploring their respective relationships with optimism and pessimism as personality traits as well as cognitive expectancies. As college students have been found to experience high levels of potentially traumatic events and high levels of negative impact related to such (Frazier et al., 2009; Walters et al., 2025), it is important to further understand how other factors, such as personality and cognitive patterns, may explain their resilient tendencies and PTG. We aimed to provide insight into how optimism and pessimism could be associated with PTG and resilience in different ways.

### 4.1. Roles of Optimism and Pessimism in PTG and Resilience

First, we hypothesized that dispositional optimism should be positively associated with PTG and resilience, as consistent with the literature, and dispositional pessimism would be negatively associated with only resilience. This hypothesis (Hypothesis 1) was supported, showing the asymmetrical findings between PTG and resiliency in terms of their respective relationship with dispositional optimism and pessimism.

We also hypothesized that, cognitively, people who believe positive events are likely to happen to them would show higher levels of resilient tendencies and PTG, whereas people who believe negative events are less likely to happen to them would show higher resilience but may or may not show PTG. As shown in Table 5, this hypothesis (Hypothesis 1) was partially supported. Of 10 positive future events, 9 of their initial estimates were positively associated with PTG, whereas only 3 of them were positively associated with resilient tendencies. On the other hand, of 10 negative future events, none of them was associated with PTG but 6 of the initial estimates for the negative future events were negatively associated with resilient tendencies. Thus, the more people believe negative events are likely to happen to them in the future, the less they show resilient tendencies, but such relationships were not found with PTG.

These asymmetrical findings demonstrate the paradoxical nature of PTG experiences (i.e., positive changes are experienced without diminishing negatives), often indicated by the coexistence of positives and negatives (Tedeschi et al., 2018). This coexistence may be demonstrated by the PTG and posttraumatic depreciation (PTD) theoretical model which presents evidence that individuals may experience negative changes following highly challenging experiences in the same contents as PTG in a curvilinear way (Taku et al., 2021). Also, these results (i.e., pessimism is negatively associated with resilience but not with PTG) may be relevant to the fact that resilience is unidimensional whereas PTG is multidimensional, meaning people with pessimistic attitudes or personality traits may experience some aspect of PTG (e.g., “I am better able to accept the way things work out”) but not the other aspects of PTG that may be more directly associated with optimistic viewpoints (e.g., “I learned a great deal about how wonderful people are”), which in turn may cancel each other out, showing no systematic relationship. Furthermore, believing that negative events are less likely to happen to oneself may demonstrate character strengths with a sense of confidence and hope for individuals with highly resilient tendencies (e.g., Akhtar et al., 2024), which could be another reason why pessimism was only associated with resilience and not with PTG.

### 4.2. Comparative Optimism and Pessimism

The second hypothesis focused on the relationships between comparative optimistic or pessimistic tendencies and PTG as well as resilience. Because previous studies in the field of PTG focused on dispositional aspects of optimism and pessimism, we could not form the specific hypotheses about the relationships between comparative optimism and PTG but we hypothesized that people who show comparative optimism would show resilient tendencies (Hypothesis 2). This hypothesis was not supported. People who showed comparative optimism for only one event (i.e., “Control my weight for at least 10 years”) reported significantly higher resilient tendencies than those who showed comparative pessimism but not for the other 19 events. It is understandable that people who show comparative optimism for this event (i.e., those who believe they can control their weight but others would not be able to) held more resilient tendencies than those who show comparative pessimism (i.e., those who believe they cannot control their weight but others can). These mindsets (i.e., comparative optimism/pessimism cognitive expectancies) were not related to PTG.

Following adversity or trauma, individuals may display some cognitive comparison biases (Gower et al., 2022) which can impact one’s beliefs or flexibility. However, in the current study, these biases were only shown in relation to resilience, but by one of twenty scenarios. The reason why only one event yielded significant differences may be the fact that we used less lenient *p*-values (e.g., if we used a 5% significance level, seven more events would have shown significant differences), and further research is needed to replicate the results. Importantly, while testing this hypothesis, individuals were categorized into three groups (i.e., those who showed comparative optimism, those who provided the same estimates, and those who showed comparative pessimism) to lend insight into overall decision making of change. We took this approach because, in our study, participants had an opportunity to provide the exact same estimates (e.g., 50% for me and 50% for others, or 30% for me and 30% for others). For example, 82 of 347 participants in fact gave the same estimates for the scenario of “Control weight for 10 years”. However, other participants gave different estimates (e.g., 50% for me and 49% for others). In such a situation, the differences between (50% for me; 49% for others) and (50% for me; 20% for others) make little difference in this study, because either way, it indicates that the participants were comparatively optimistic, given that they could have provided 50–50. If we relied on differences scores, extreme cases (e.g., 50% for me; 10% for others) will have more influences on the results and the estimates close to each other, e.g., 50% for me; 49% for others will be statistically closer to those who showed zero difference, even though subjective meanings between people who gave different estimates (50–49%) and those who gave the same estimates (50–50%) are different from each other. With this reason, we used categorization; however, future studies should further explore the roles of comparative optimism and pessimism in PTG and resilience by using different analytical methodologies.

### 4.3. Belief Updating

The third hypothesis focused on the roles of belief updates in PTG and resilience. As for the second hypothesis, it was not possible to make hypotheses in specific directions because, theoretically, individuals who are willing to make adjustments in their beliefs when they obtain new information about average people may show a higher level of resilient tendencies due to their cognitive flexibility, but they may also feel that it is unnecessary to make such adjustments due to their resilient tendencies that are associated with capability, competence, and a sense of confidence. Similarly, without formulating specific hypotheses, we explored the roles of belief updates in PTG in the current study, assuming that belief updates may be irrelevant to PTG but more relevant to resilience.

We once again used group-based analyses, shown in Table 3, to gain a basic understanding of the directional patterns. Our results yielded mixed findings. Of 20 events, only one event showed significant differences in resilience. Individuals with higher optimistic tendencies who chose to “stick to” their beliefs held more resilient tendencies than those who were initially displaying more pessimistic tendencies with or without making changes. This result indicates that, when the event is something they can control (i.e., “Control my weight for at least 10 years”), the absolute level of optimism rather than flexible cognitive adjustments plays a larger role in resilient tendencies. On the other hand, of twenty events, two showed significant differences in PTG. Regarding the first event (i.e., “Never have any financial problems for the rest of life”), PTG was higher in those who were initially optimistic but then adjusted to be modest (i.e., lowered their estimates when they reviewed the new information for average people) than those who were initially pessimistic but adjusted to match the actual likelihood. Similarly, regarding a positive event that is somewhat ambiguous (i.e., “Live with partner happily ever after”), PTG was higher in those who were initially optimistic but then adjusted to be modest than those who were initially pessimistic with or without having adjusted. These results indicate that those who display cognitive flexibilities when they realized that their initial estimation was too optimistic were more likely to report PTG. Such realizations and adjustments might have helped some people to deliberately reflect on how they changed after adversity, but such speculation needs to be further tested in future studies.

### 4.4. Implications

Today, the concept of resilience has been established as one of the desirable abilities and traits that humans should have (Rutter, 2007), as it was frequently demonstrated as a useful personal resource (e.g., Raper et al., 2023; Smith et al., 2010). Many training programs, therapies, and psycho-educational interventions that aim to increase resilient tendencies have been proposed and developed with various targets, including cancer patients (Ding et al., 2024) and caregivers (Wu et al., 2024). However, the current results indicate that resilience may include not just adaptive elements but potentially maladaptive aspects, such as a sense of overconfidence (Kleiman et al., 2017). On the other hand, the concept of PTG, too, has been recognized as a desirable path and outcome after experiencing a highly stressful and potentially traumatic life event (e.g., Moore et al., 2021), but this perception was not fostered by perceiving negative events to be less likely to happen to them. Such results may imply the potential role of defensive pessimism.

Moreover, PTG, but not resilience, was higher in those who showed flexibility to be modest (i.e., belief updates) when they realized that they were initially too optimistic. Realistically adjusting one’s cognitive beliefs may be an important ability and coping strategy. It has been suggested that individuals with optimistic tendencies are highly adaptive and flexible (Conversano et al., 2010; Salah et al., 2022); therefore, more research is needed to further understand how such flexibility-related cognitive ability may be associated with PTG and resilience, as cognitive flexibility has been more widely studied among defensive pessimists (Pérès et al., 2002; Tekin et al., 2022) than dispositional pessimists.

### 4.5. Limitations

Though the current study provided insight into the relationship that optimism and pessimism may have with PTG and resilience by looking at different aspects (i.e., dispositional personality traits as well as cognitive expectancies), it is not without limitations. First, the current study held a lack of diversity with a primarily white, female, and young adult psychology student sample. Gender and other demographic information, such as specific religious or spiritual beliefs, was not included in the analyses. Even though demographic factors such as gender differences in PTG have been found to be relatively small (Vishnevsky et al., 2010) and may not have a substantial impact on findings, future studies should consider addressing the impact of demographics and possible confounding variables. It is also important to note that the psychology student sample we targeted in this study might have posed large limitations due to their enhanced education in understanding the underpinnings of psychological research processes. This may in turn have impacted their responses during the tasks. The lack of diversity and potential educational influence provides difficulty in generalizing the current results to other populations. Second, the specific events or recency within five years under which PTG occurred was not accounted for in the analysis, thus limiting a nuanced understanding of PTG. Third, the current study utilized 20 hypothetical life events to measure cognitive expectations, and we investigated each event separately, instead of grouping them together, when testing Hypotheses 2 and 3. We chose this approach because we thought the nature of the event itself, even if they all imply positive (e.g., “Never have any financial problems for the rest of life”, “Receive an A in a class”) or negative events (e.g., “Develop a brain tumor”, “Get robbed”), should influence the relationship with PTG and resilience in different ways.

Nevertheless, researchers should consider grouping events and using the Belief Update Task (Sharot et al., 2011) exactly as the creators had intended. We did not ask participants about their perceptions of, for example, the positive or negative valence of each event or their past experiences related to each event. Additionally, social desirability bias in cognitive tasks may be a potential confounder of the data, although precautions such as anonymity (i.e., paper-and-pencil survey) and clear-cut instructions with minimally described estimations were taken. Due to these missing pieces of information, we could not control the participants’ prior experiences, cognitive appraisals, and potential biases when analyzing data and testing the hypotheses as well as interpreting the results.

### 4.6. Future Directions

Researchers recommend using, for example, 10 to 70% rather than 0 to 100% when estimating the likelihood of each event to allow study participants to update their beliefs without being affected by ceiling or floor effects (Sharot & Garrett, 2022). This is because the assumption is, if the participants do not update their beliefs, then it is the researcher’s fault for not describing the cognitive task appropriately to allow study participants to update their beliefs (i.e., healthy individuals must be able to update their beliefs). The current study questioned this assumption, because we thought some individuals may believe that something is nearly certain to happen and the choice of “no update” should show an important quality of individual differences. Nevertheless, the current study failed to assess “why” people did not change or changed their estimates when they reviewed the novel information. Such reasons may further help researchers to better understand the characteristics of people who hold such cognitive expectancies, which in turn helps us to better explain how cognitive expectancies may affect PTG and resilient tendencies.

In addition, future research could use more comprehensive measurement assessments, such as the Posttraumatic Growth Inventory—Expanded Version (PTGI-X) which captures greater existential–spiritual dimensions of PTG (Tedeschi et al., 2018). Furthermore, pessimism has been explored with negative life outcomes (Whitfield et al., 2020), as people with pessimistic tendencies may hold a unique adaptive strategy of belief adjustment in the face of certain life events. Aiming to further understand the potential cognitive flexibility of individuals with pessimistic tendencies and how cognitive flexibility could work in providing positive life outcomes may reveal a silver lining with a group often thought of as high risk for negative life outcomes. Finally, the current study is cross-sectional and relies on self-report; thus cause and effect could not be determined.

## 5. Conclusions

The current study explored distinctions between posttraumatic growth and resilience in relation to dispositional optimism, dispositional pessimism, and cognitive expectancies related to optimism/pessimism. As optimism and pessimism have largely been examined in the literature as dispositional traits, where the current study’s findings of associations between PTG, resilience, dispositional optimism, and dispositional pessimism produced similar findings to that of previous research, less is known about the roles of optimism and pessimism as cognitive patterns in PTG and resilience. Results revealed that higher amounts of optimism as a cognitive expectancy for positive events were associated with PTG, whereas lower amounts of pessimism as a cognitive expectancy for negative events, as well as comparative optimism for one positive event, were associated with resilience, thus revealing potentially distinct characteristics of PTG and resilience. Such findings may suggest resilience reflects non-negativity and positivity within social comparisons (i.e., having less cognitively pessimistic attitudes toward future events and more optimistic attitudes by comparing with others), whereas PTG may reflect hope only (i.e., being able to be optimistic for the future for oneself, but not for others, and holding pessimistic perspectives or not) in the context of expecting and adjusting for the likelihood of future life events. Future studies may seek to further and more comprehensively examine these conceptual differences, particularly advancing conceptualization of optimism and pessimism as cognitive patterns rather than only dispositional traits, to help concretely inform actionable and applied implications of this research to further investigate potential complementary associations of PTG and resilience.

## Figures and Tables

**Table 1:** Demographics of Sample (*N* = 347).

	*n*	%
Gender		
Female	278	80.1
Male	68	19.6
Prefer not to answer	1	0.3
Race		
White	207	59.7
African American	57	16.4
Asian	19	5.5
Middle Eastern heritage	35	10.1
Mixed	14	4
Latino Hispanic or Latin American heritage	11	3.2
Native Hawaiian or other Pacific Islander	1	0.3
American Indian or Alaskan Native	1	0.3
Other	2	0.6
Religion		
Christianity	254	73.2
Judaism	6	1.7
Islamic	14	4
Buddhism	2	0.6
Agnostic	22	6.3
Unsure	21	6.1
Other	24	6.9
Type of Potentially Traumatic Life Events		
Accident or injury	22	6.3
Serious illness	24	6.9
Serious school/academic problems	18	5.2
Family issues	54	15.6
Financial or work-related issues	18	5.2
Death of someone close	103	29.7
Assault on you or someone you know	12	3.5
Moving residence or changing schools	23	6.6
Bullying or abuse	8	2.3
Friendship problems	17	4.9
Romantic relationship problems	32	9.2
Other	7	2.0
Unsure	9	2.6

**Table 2:** Three Estimates for the Positive and Negative Future Life Events and Corresponding Given “Actual” Likelihoods.

	Future Life Scenario	*M* (*SD*) of the First Estimate	*M* (*SD*) of the Second Estimate	Given Likelihood	*M* (*SD*) of the Third Estimate
1	Receive an A in a class (+)	83.73 (17.94)	80.60 (17.68)	66%	77.45 (18.35)
2	Fall in love (+)	76.37 (26.82)	82.45 (17.31)	93%	82.13 (23.66)
3	Develop a brain tumor (−)	25.98 (22.51)	28.71 (22.44)	31%	23.33 (13.68)
4	Live to 90 years of age (+)	50.75 (27.11)	52.24 (23.78)	24%	29.49 (21.08)
5	Fail a course (−)	29.90 (29.54)	41.50 (24.31)	21%	18.34 (18.43)
6	Go bankrupt (−)	17.98 (20.46)	33.13 (24.09)	39%	20.07 (14.11)
7	Never have any financial problems for the rest of life (+)	25.85 (27.29)	28.85 (25.33)	30%	25.06 (18.06)
8	Get fired from job (−)	22.49 (24.25)	36.66 (23.50)	13%	12.82 (14.72)
9	Lose a friend (−)	63.73 (30.61)	64.67 (27.52)	76%	66.93 (23.92)
10	Get a job directly after college (+)	65.49 (24.53)	61.91 (19.97)	56%	61.03 (20.42)
11	Win more than $10,000 in the lottery (+)	7.70 (15.00)	13.76 (18.79)	24%	11.81 (11.45)
12	Win a raffle with an expensive prize (+)	16.16 (19.57)	22.58 (22.15)	12%	12.19 (12.12)
13	Get cheated on (−)	36.69 (29.83)	47.27 (24.53)	23%	25.06 (21.76)
14	Heart attack before age 70 (−)	30.57 (23.48)	37.05 (21.52)	60%	42.53 (21.34)
15	Never be successful at school (−)	11.79 (18.26)	24.56 (20.43)	11%	8.49 (12.13)
16	Get robbed (−)	24.56 (20.43)	29.54 (20.76)	33%	23.72 (13.66)
17	Find $20 on the street (+)	29.44 (26.81)	33.28 (26.20)	56%	37.68 (20.61)
18	Struck by lightning (−)	5.78 (11.84)	8.70 (16.70)	4%	3.10 (4.39)
19	Live with partner happily ever after (+)	67.43 (28.68)	63.35 (22.77)	63%	68.71 (22.76)
20	Control my weight for at least 10 years (+)	63.16 (31.83)	54.87 (22.49)	82%	71.34 (25.00)

Note. (+) positive life scenarios, (−) negative life scenarios. The first estimate shown in the third column indicates the likelihood that each of these events will happen to oneself. The second estimate shown in the fourth column indicates the likelihood that each event will happen to others of same age/gender. The third estimate shown in the sixth column indicates the likelihood that the participants reevaluate after seeing the “actual prevalence” rates shown in the fifth column.

**Table 3:** Means, standard deviations, and correlations among study variables.

Variables	α	*M*	*SD*	1	2	3
1. PTG	0.85	3.20	1.04	-		
2. Resilience	0.86	3.05	0.83	−0.02	-	
3. Dispositional Optimism	0.71	3.31	0.91	0.32 **	0.33 **	-
4. Dispositional Pessimism	0.81	2.75	0.98	−0.10	−0.36 **	−0.58 **

Note. ** *p* < 0.01.

**Table 4:** Multiple regression analyses explaining the roles of dispositional optimism and pessimism in PTG and resilience.

Independent Variables	PTG		Resilience	
	*β*	*SE*	*β*	*SE*
1. Dispositional Optimism	0.40 **	0.07	0.18 **	0.06
2. Dispositional Pessimism	0.14	0.07	−0.26 **	0.05

Note. ** *p* < 0.01.

**Table 5:** Correlations between the first estimate of each scenario and study variables.

	Future Life Scenario	PTG	Resilience	Dispositional Optimism	Dispositional Pessimism
1	Receive an A in a class (+)	0.11 *	0.16 **	0.21 **	−0.22 **
2	Fall in love (+)	0.19 **	0.01	0.20 **	−0.20 **
3	Develop a brain tumor (−)	0.03	−0.05	−0.14 *	0.07
4	Live to 90 years of age (+)	0.11 *	0.05	0.27 **	−0.20 **
5	Fail a course (−)	0.04	−0.07	−0.08	0.12 *
6	Go bankrupt (−)	0.04	−0.11	−0.11 *	0.19 **
7	Never have any financial problems for the rest of life (+)	0.18 **	0.09	0.24 **	−0.17 **
8	Get fired from job (−)	−0.02	−0.12 *	−0.11 *	0.12 *
9	Lose a friend (−)	−0.09	−0.11 *	−0.03	0.04
10	Get a job directly after college (+)	0.20 **	0.13 *	0.27 **	−0.21 **
11	Win more than $10,000 in the lottery (+)	0.17 **	0.01	0.12 *	−0.08
12	Win a raffle with an expensive prize (+)	0.17 **	0.12 *	0.17 **	−0.21 **
13	Get cheated on (−)	0.04	−0.16 **	−0.13 *	0.18 **
14	Heart attack before age 70 (−)	−0.01	−0.11 *	−0.21 **	0.19 **
15	Never be successful at school (−)	−0.04	−0.18 **	−0.21 **	0.23 **
16	Get robbed (−)	−0.05	−0.15 **	−0.16 **	0.19 **
17	Find $20 on the street (+)	0.13 *	0.05	0.10	−0.13 *
18	Struck by lightning (−)	0.-00	−0.11	−0.06	0.10
19	Live with partner happily ever after (+)	0.21 **	0.01	0.31 **	−0.23 **
20	Control my weight for at least 10 years (+)	0.08	0.19 **	0.27 **	−0.23 **

Note. * *p* < 0.05, ** *p* < 0.01.

**Table 6:** Means, standard deviations, and one-way ANOVA results of Event 7 (“Never have any financial problems for the rest of life”).

Variables	Group 1	Group 2	Group 3	Group 4	*F*	*p*
Sample size	(*n* = 95)	(*n* = 170)	(*n* = 12)	(*n* = 49)		
First estimate	60.35 (18.52) ^a^	8.71 (11.35) ^b^	58.33 (20.38) ^a^	7.98 (8.45) ^b^	331.16	0.001
Third estimate	30.26 (19.28) ^a^	23.83 (13.53) ^b^	58.33 (20.38) ^c^	7.98 (8.45) ^d^	44.07	0.001
PTG	3.51 (0.90) ^a^	3.07 (1.05) ^b^	3.49 (0.97)	2.96 (1.16) ^b^	5.06	0.002
Resilience	3.02 (0.81)	3.09 (0.82)	3.25 (0.78)	2.84 (0.88)	1.46	0.23

Note. The values with different superscript letters are significantly different at *p* < 0.05, per Tukey post hoc analyses; Group 1 = initially optimistic and adjusted estimate to be lower, Group 2 = initially pessimistic and adjusted estimate to be higher, Group 3 = initially optimistic and made no adjustment, Group 4 = initially pessimistic and made no adjustment.

**Table 7:** Means, standard deviations, and one-way ANOVA results of Event 19 (“Live with partner happily ever after”).

Variables	Group 1	Group 2	Group 3	Group 4	*F*	*p*
Sample size	(*n* = 126)	(*n* = 100)	(*n* = 83)	(*n* = 33)		
First estimate	86.17 (9.69) ^a^	34.07 (20.05) ^b^	88.78 (10.73) ^a^	42.73 (15.67) ^c^	349.59	0.001
Third estimate	73.83 (15.23) ^a^	55.87 (21.27) ^b^	88.02 (13.85) ^c^	42.73 (15.67) ^d^	84.48	0.001
PTG	3.39 (0.97) ^a^	3.01 (1.08) ^b^	3.27 (1.04)	2.86 (1.04) ^b^	4.06	0.007
Resilience	3.02 (0.78)	3.06 (0.78)	3.06 (0.93)	3.06 (0.89)	0.05	0.98

Note. The values with different superscript letters are significantly different at *p* < 0.05 per Tukey post hoc analyses; Group 1 = initially optimistic and adjusted estimate to be lower, Group 2 = initially pessimistic and adjusted estimate to be higher, Group 3 = initially optimistic and made no adjustment, Group 4 = initially pessimistic and made no adjustment.

**Table 8:** Means, standard deviations, and one-way ANOVA results of Event 20 (“Control my weight for at least 10 years”).

Variables	Group 1	Group 2	Group 3	Group 4	*F*	*p*
Sample size	(*n* = 53)	(*n* = 169)	(*n* = 70)	(*n* = 52)		
First estimate	93.13 (12.11) ^a^	42.44 (26.46) ^b^	94.41 (5.52) ^a^	56.65 (24.02) ^c^	137.91	0.001
Third estimate	81.72 (17.92) ^a^	63.59 (23.94) ^b^	94.41 (5.52) ^c^	55.88 (24.06) ^b^	51.36	0.001
PTG	3.26 (1.11)	3.18 (1.02)	3.28 (1.06)	3.06 (1.02)	0.52	0.67
Resilience	3.20 (0.84) ^ab^	2.95 (0.79) ^a^	3.30 (0.81) ^b^	2.87 (0.85)	4.48	0.004

Note. The values with different superscript letters are significantly different at *p* < 0.05 per Tukey post hoc analyses; Group 1 = initially optimistic and adjusted estimate to be lower, Group 2 = initially pessimistic and adjusted estimate to be higher, Group 3 = initially optimistic and made no adjustment, Group 4 = initially pessimistic and made no adjustment.

## Data Availability

The data is available on Open Science Framework: https://osf.io/z2evr/ accessed on 23 April 2025.
